# Role of Alanine Racemase Mutations in Mycobacterium tuberculosis d-Cycloserine Resistance

**DOI:** 10.1128/AAC.01575-17

**Published:** 2017-11-22

**Authors:** Yoshio Nakatani, Helen K. Opel-Reading, Matthias Merker, Diana Machado, Sönke Andres, S. Siva Kumar, Danesh Moradigaravand, Francesc Coll, João Perdigão, Isabel Portugal, Thomas Schön, Dina Nair, K. R. Uma Devi, Thomas A. Kohl, Patrick Beckert, Taane G. Clark, Gugu Maphalala, Derrick Khumalo, Roland Diel, Kadri Klaos, Htin Lin Aung, Gregory M. Cook, Julian Parkhill, Sharon J. Peacock, Soumya Swaminathan, Miguel Viveiros, Stefan Niemann, Kurt L. Krause, Claudio U. Köser

**Affiliations:** a Department of Microbiology and Immunology, Otago School of Medical Sciences, University of Otago, Dunedin, New Zealand; b Maurice Wilkins Centre for Molecular Biodiscovery, The University of Auckland, Auckland, New Zealand; c Department of Biochemistry, Otago School of Medical Sciences, University of Otago, Dunedin, New Zealand; d Molecular and Experimental Mycobacteriology, Research Center Borstel, Borstel, Germany; e German Center for Infection Research, Partner Site Hamburg-Borstel-Lübeck, Germany; f Unidade de Microbiologia Médica, Global Health and Tropical Medicine, Instituto de Higiene e Medicina Tropical, Universidade Nova de Lisboa, Lisbon, Portugal; g Division of Mycobacteriology (National Tuberculosis Reference Laboratory), Research Center Borstel, Borstel, Germany; h National Institute for Research in Tuberculosis, Chennai, India; i Wellcome Trust Sanger Institute, Hinxton, United Kingdom; j London School of Hygiene and Tropical Medicine, London, United Kingdom; k Med.ULisboa–Instituto de Investigação do Medicamento, Faculdade de Farmácia, Universidade de Lisboa, Lisbon, Portugal; l Department of Clinical and Experimental Medicine, Division of Medical Microbiology, Linköping University, Linköping, Sweden; m Department of Clinical Microbiology and Infectious Diseases, Kalmar County Hospital, Kalmar, Sweden; n National Reference Laboratory, Ministry of Health, Mbabane, Swaziland; o National Tuberculosis Control Program, Ministry of Health, Manzini, Swaziland; p Institute of Epidemiology, University Hospital Schleswig-Holstein, Campus Kiel, Kiel, Germany; q Tartu University Hospital, United Laboratories, Mycobacteriology, Tartu, Estonia; r Department of Medicine, University of Cambridge, Cambridge, United Kingdom; s Department of Health Research and Director General, Indian Council of Medical Research, New Delhi, India; t Department of Genetics, University of Cambridge, Cambridge, United Kingdom

**Keywords:** Mycobacterium tuberculosis, cycloserine, alanine racemase

## Abstract

A screening of more than 1,500 drug-resistant strains of Mycobacterium tuberculosis revealed evolutionary patterns characteristic of positive selection for three alanine racemase (Alr) mutations. We investigated these mutations using molecular modeling, *in vitro* MIC testing, as well as direct measurements of enzymatic activity, which demonstrated that these mutations likely confer resistance to d-cycloserine.

## TEXT

In 2015, the Global Drug Facility declared that the cost of d-cycloserine (DCS), a group C drug to treat tuberculosis (TB), would be cut by more than half to as little as $0.19 per capsule to support the treatment of multidrug-resistant (MDR) and extensively drug-resistant (XDR) TB, which represent a major threat to public health (1). In light of this announcement, a better understanding of the resistance mechanisms to this drug is required to facilitate phenotypic as well as genotypic drug susceptibility testing (DST), both in the context of surveillance and individual patient treatment, to avoid the severe side effects of this drug (2, 3).

Studies of the mode of action of DCS in mycobacteria have produced contradictory results, with some studies pointing to alanine racemase (Alr) as the primary target and others supporting d-alanine–d-alanine ligase (DdlA) (4–9). However, molecular data from Mycobacterium tuberculosis complex (MTBC) have implicated only *alr* in DCS resistance, which can also be conferred by mutations in alanine dehydrogenase (*ald*) or a permease (*cycA*) gene (10, 11). Using molecular modeling, we had predicted that the *alr* M319T mutation observed in an XDR strain would likely confer resistance to DCS, which was subsequently confirmed by Desjardins et al. using the unrelated strain TKK_04_0105 (see Table S1 in the supplemental material) (2, 11). Desjardins et al. described a number of additional *alr* mutations in strains with elevated DCS MICs, including a C-to-T nucleotide change 8 bp upstream of the experimentally confirmed start codon of *alr* (strain TKK_02_0050 in Table S1) (11, 12). This was notable, as Merker et al. had previously reported that, compared with the susceptible parental *alr* wild-type strain, the acquisition of this mutation during treatment with DCS correlated with DCS resistance, which suggested that *alr* mutations might be both necessary and sufficient to confer DCS resistance (13).

**FIG 1:**
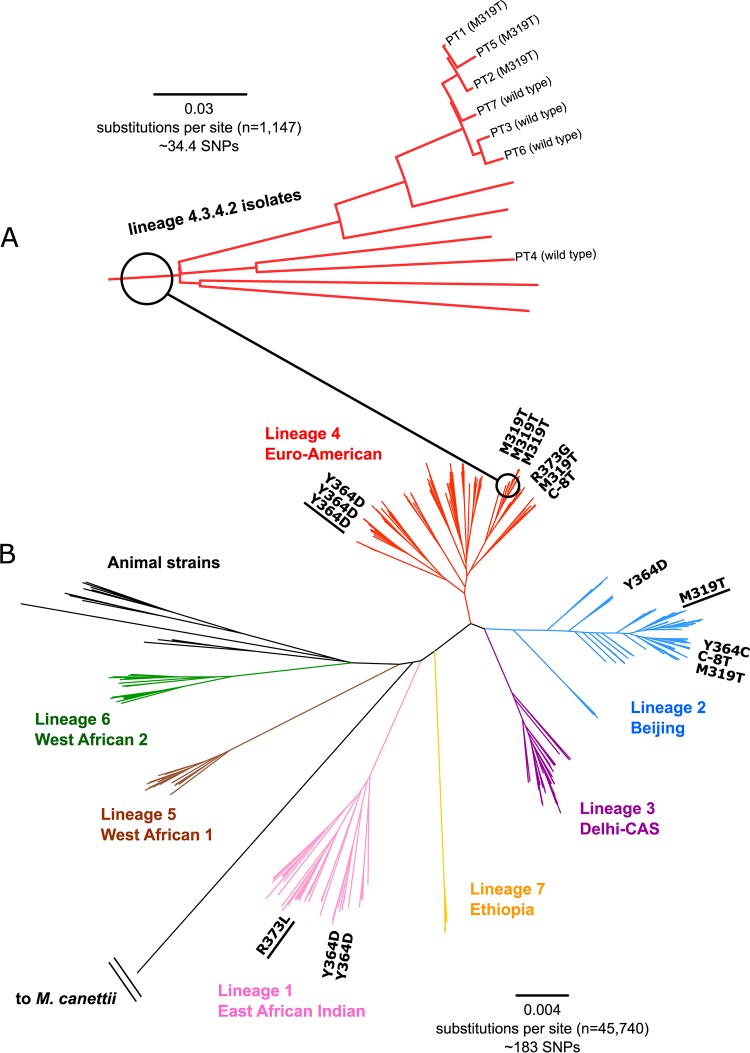
Maximum likelihood tree based on a concatenated sequence alignment of 45,740 variable sites (1,000 resamplings, general time-reversible [GTR] nucleotide substitution model) showing the *alr* mutants from Table S1 in the context of a globally representative reference collection of 287 MTBC strains. (A) Zoomed-in part of the overall tree (B), showing the phylogenetic relationship between the three Portuguese M319T mutants (PT1, PT5, and PT2) and the control strains (PT7, PT3, PT6, and PT4) tested in MGIT and Sensititre. The three Indian M319T, R364D, and R373G mutants that were tested with the 1% proportion LJ method in this study are underlined. The C-8T, M319T, and R364D mutations were homoplastic (i.e., they were acquired multiple times independently) and two different amino acid changes were observed at codon 373 (i.e., R373L and R373G). Thus, all mutations show evolutionary patterns of positive selection. SNPs, single-nucleotide polymorphisms; CAS, central Asian strain.

To gain further insights into the impact of *alr* mutations, we first confirmed that the aforementioned *alr* C-8T promoter mutant that evolved during treatment correlated in MICs above the current World Health Organization (WHO)-endorsed critical concentration (CC) of 30 μg/ml using the 1% proportion method on Löwenstein-Jensen (LJ) (strains PBm0 and PBm14 in Table S1; Desjardins et al. and Merker et al. had used 10% as the critical proportion and therefore had not adhered to the current WHO recommendations [11, 13, 14]). Using the same method, we also showed that two strains with *alr* M319T or Y364D mutations from XDR TB patients with a treatment history with DCS had MICs above the CC (Table S1). Moreover, we observed the M319T mutation in three XDR strains (PT1, PT2, and PT5) from Lisbon, Portugal (15). Although no CC exists for MGIT 960, this mutation correlated in an MIC increase from 16 to 64 μg/ml compared with three closely related wild-type control strains (PT3, PT6, and PT7) and one more distantly related control strain (PT4), which supported the role of this mutation in DCS resistance (Fig. 1A; Table S1). In contrast, no or minimal MIC increases were recorded when testing these Portuguese strains using Sensititre MycoTB plates (Table S1) (16). Finally, a pre-XDR *alr* R373L mutant from a patient with DCS exposure, which also harbored a deletion in *ald*, tested resistant on LJ using the 1% proportion method (Tables S1 and S2).

To study the importance of the C-8T, M319T, Y364D, and R373L mutations from an evolutionary perspective, we screened previously published and unpublished genomes of more than 1,500 MDR strains (mostly from Germany, eastern Europe, and Swaziland), which identified eight additional strains with mutations at these *alr* positions or codons (Table S1). Interrogating the genomes of these 17 strains in the context of a phylogenetically diverse reference collection that included all major MTBC lineages and species showed that the mutations had either been acquired multiple times independently and/or that different amino acid changes were present at the same codons (Fig. 1B). These mutation patterns are typically a signal of positive selection, which could have occurred in response to DCS exposure.

Molecular modeling of these coding mutations supported this hypothesis. Alr functions as a homodimer, aided by the cofactor pyridoxal 5′-phosphate (PLP) to which it is covalently bound. DCS inhibits Alr irreversibly by covalently bonding to PLP (4). We generated and analyzed a model of the complex between the M. tuberculosis Alr and DCS (Alr_*Mtb*_-DCS) (Fig. S1) (4, 17). Amino acid residues 319 and 364 were located directly in the active site (Fig. S1B). M319T was positioned close enough to allow interaction with the DCS moiety, which, given the large change of the character of the side chain, could strongly affect DCS reactivity (Fig. S1C). Y364 is involved in the positioning of the phosphate moiety of PLP and thus represents a prominent active site residue in the conserved inner layer of the substrate entrance corridor of Alr (Fig. S1B) (17). A mutation to aspartic acid introduced a shorter and negatively charged side chain, which could potentially affect PLP orientation in the active site (Fig. S1C). Moreover, it could influence DCS uptake through alteration of the entrance corridor. Interestingly, M319 is located near Y364 and, as a result, it is possible that the M319T mutation could alter the interaction with Y364, thereby affecting DCS inhibition. In contrast, the R373L mutation was not directly located within the active site but near the dimer interface and close to residues M319 and D320, which play an important role in the makeup of the active site (Fig. S1B). Consequently, the replacement of arginine with the short and hydrophobic side chain of leucine might disrupt molecular interactions at the dimer interface as well as destabilize the DCS binding site (Fig. S1C).

**FIG 2:**
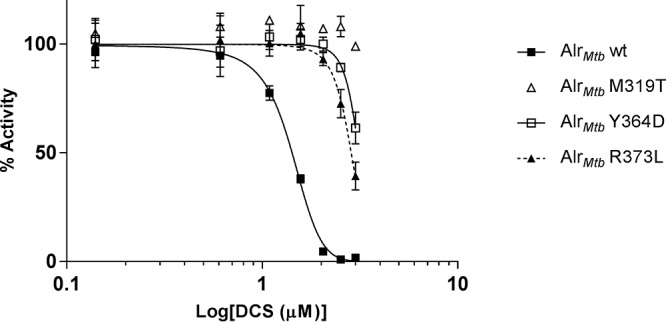
Determination of DCS IC_50_ for wild-type (wt) Alr_*Mtb*_ and the M319T, Y364D, and R373L mutants. The activity was normalized against a control with no DCS present in the assay mixture. The activity assay at each concentration was performed in triplicate, resulting in the error bars, which represent the 95% confidence interval. A variable slope model was fitted to determine the IC_50_s, which were 26.4 ± 1.7, 1,328.0 ± 340.0, and 712.0 ± 138.5 μM for the wild-type, Y364D, and R373L enzymes, respectively. The inhibition of M319T was too weak to allow for IC_50_ determination.

To test these predictions experimentally, we expressed and purified the aforementioned Alr_*Mtb*_-coding mutants, along with wild-type Alr_*Mtb*_, and determined their half-maximal inhibitory concentration (IC_50_) to measure the effectiveness of inhibition by DCS (Fig. 2). The IC_50_ for wild-type Alr_*Mtb*_ was 26.4 ± 1.7 μM, which was in the range previously reported for this compound (18, 19). From our structure-based analysis, we expected the two mutations located in the active site to show the greatest effect on DCS inhibition. Indeed, the M319T mutant enzyme showed minimal inhibition by DCS, even at 1,000 μM (Fig. 2). Thus, the IC_50_ of this mutant could not be determined. The IC_50_ of the Y364D mutant showed a 50-fold increase to 1,328.0 ± 340.0 μM. The R373L mutation, which was not located directly within the active site, also showed a significant increase in resistance to DCS, with an IC_50_ of 712.0 ± 138.5 μM (27-fold increase).

Taken together, these data suggested that *alr* mutations likely confer DCS resistance, although allelic exchange experiments are required to formally prove this (particularly for R373L, which coincided with a deletion in *ald* and, consequently, may not be sufficient to confer resistance on its own). Although the relationship between MICs and IC_50_s can be complex, the observation that MICs increased by only 4- to 16-fold versus at least 25-fold increases for IC_50_s supports the notion that DCS inhibits multiple targets, as noted earlier. This study should be complemented with extensive MIC testing of phylogenetically diverse pansusceptible MTBC strains to define the epidemiological cutoff value, given that it is unclear based on which evidence the current WHO CC on LJ has been set (3, 14, 20, 21). Moreover, further MIC testing of likely DCS-resistant strains is needed to investigate whether the Sensititre system is less reliable at detecting DCS resistance than are LJ and MGIT. Finally, the impact of *alr* mutations on resistance on terizidone remains to be investigated.

## Supplementary Material

Supplemental material
