# Supplementary material for: Role of Alanine Racemase Mutations in Mycobacterium tuberculosis d-Cycloserine Resistance
Source: Antimicrob Agents Chemother. 2017 Nov 22;61(12):e01575-17. doi: 10.1128/AAC.01575-17 (PMC5700341; doi:10.1128/AAC.01575-17)
Supplement: Supplemental material [file supp_61_12_e01575-17__index.html]

Supplemental material 

# Role of Alanine Racemase Mutations in Mycobacterium tuberculosis d-Cycloserine Resistance

## Supplemental material

- Supplemental file 1 -

  Supplemental material

  PDF, 3.3M
