# Supplementary material for: Role of Alanine Racemase Mutations in Mycobacterium tuberculosis d-Cycloserine Resistance
Source: Antimicrob Agents Chemother. 2017 Nov 22;61(12):e01575-17. doi: 10.1128/AAC.01575-17 (PMC5700341; doi:10.1128/AAC.01575-17)
Supplement: Supplemental material [file AAC.01575-17_zac012176719s1.pdf]

Materials and Methods

MIC testing in Germany and India

Löwenstein-Jensen (LJ) with a 1% critical proportion at 1.875, 3.75, 7.5, 10, 15, 20, 30, 40, 60, 120, and 240 µg/ml was used for MIC testing.

MIC testing in Portugal

DCS MICs were initially determined using Sensitrite MycoTB plates (Trek Diagnostics, Thermo Fisher Scientific, Oakwood Village, USA). Briefly, the *M. tuberculosis* strains were grown on LJ or Middlebrook 7H9 (MB7H9) supplemented with 10% OADC (oleic acid, albumin, dextrose, and catalase) (Becton and Dickinson, Sparks, MD, USA). After 2-3 weeks of growth on LJ, colonies were added to a test tube containing a saline-tween 80 solution and glass beads. The suspension was vortexed and the concentration adjusted to a turbidity of a McFarland standard of 0.5. Thereafter, the bigger clumps were allowed to settle for 15 min. 100 µl of this suspension were transferred to 11 ml of MB7H9 plus 10% OADC to give a final inoculum of approx. 10^5 cells/ml and mixed by vortexing. 100 µl of this suspension were added to each of the 96-wells of the MycoTB plate. For strains grown in MB7H9, these were allowed to grow until an OD600nm of 0.8 at 37ºC. The inoculum was prepared by diluting these cultures in MB7H9/OADC medium to a final density of approx. 10^5 cells/ml, of which 100 µl were added to each of the 96-wells of the MycoTB plate. All the plates were covered, sealed in plastic bags, and incubated at 37ºC during 10 days. After this period, the plates were checked for growth. The MIC was defined as the lowest concentration of antibiotic that inhibited bacterial growth completely. When growth in the growth control-well was insufficient, the plates were re-incubated and re-read at day 21.

Drug susceptibility testing (DST) for DCS was conducted using the BACTEC MGIT 960 system and Epicenter V5.80A/TB eXIST (Becton and Dickinson). DCS (Sigma Aldrich, St. Louis, MO, USA) was tested at 2, 4, 8, 16, 32, and 64 µg/ml. To obtain primary cultures for DST, the strains were grown in MGIT tubes until they reached 100-200 growth units (GU). For DST, the MGIT tubes were inoculated with 0.8 ml of SIRE supplement (Becton Dickinson), 0.1 ml of a solution of DCS to achieve the desired final concentration, and 0.5 ml of the suspension of the strain. For the preparation of the drug-free proportional control, the strain suspension was diluted 1:100 and 0.5 ml inoculated in the tube. To monitor the normal growth of the strain, an absolute control was prepared using 500 µl of the undiluted strain suspension as inoculum. The interpretation of the results was performed as previously described (1, 2). Briefly, when growth was observed in the drug-containing tube (GU≥100) before the proportional growth control (1% inoculum) was positive (GU=400), this indicated that more than 1% of the population was growing in the presence of the drug and as per proportion testing, the strain was considered resistant at the corresponding drug concentration. The MIC was considered as the lowest concentration with GU <100 when the drug-free proportional control tube reached the positivity threshold of 400 GU.

WGS and bioinformatics analyses

Strains listed in Table S1 were classified using the nomenclature by Coll et al. and analyzed in the context of a reference collection that encompassed all seven *M. tuberculosis* and *M. africanum* lineages, as well as animal strains and *M. canettii* (3-5). WGS was performed using Illumina Technology (MiSeq and HiSeq 2500) with Nextera XT library preparation kits as instructed by the manufacturer (Illumina). By contrast, the NEXTflex DNA Sequencing Kit (Bioo Scientific) was used to prepare the Indian strains for sequencing on an Illumina MiSeq.

Fastq files/reads were mapped to the *M. tuberculosis* H37Rv genome (GenBank ID: NC_000962.3) with BWA (6). Alignments were refined with GATK and Samtools toolkits with regard to base quality re-calibration, alignment corrections for possible PCR and insertions and deletions indel artefact (7, 8). Variants were extracted with customized perl scripts employing thresholds of a minimum

coverage of 4 reads in both forward and reverse orientation, 4 reads calling the allele with at least a phred score of 20, and 75% allele frequency.

In the combined dataset, we allowed 5% of all samples to fail the aforementioned threshold criteria in individual genome positions to compensate for coverage fluctuations in certain genome regions. Overall, regions annotated as 'repetitive' elements (e.g. PPE and PE-PGRS gene families), resistance associated genes, indels, and consecutive variants in a 12 bp window (putative artefacts flanking indels were excluded. The remaining single nucleotide polymorphisms (SNPs) were considered as valid and used for concatenated sequence alignments.

The maximum likelihood tree was calculated with FastTree using the concatenated sequence alignment, a general time reversible (GTR) nucleotide substitution model, 1,000 resamples and Gamma20 likelihood optimization to account for evolutionary rate heterogeneity among sites. Alr mutations discussed in this manuscript were mapped on the corresponding branches/sub-groups (9).

Site-direct mutagenesis of Alr_{Mtb}

A one-step site-directed mutagenesis protocol was employed to the generate M319T, Y364D and R373L Alr_{Mtb} expression constructs (10). Mutagenesis PCR was performed using pMB2103 (a pET28 vector with a hexa-histidine tag at the N-terminal end and wild-type Alr_{Mtb} (11)) as a template with the primers shown in Table S3. PCR mixes were prepared according to the manufacturer's recommendation (FINNZYMES) using Phusion™ DNA polymerase. The initial denaturation step was for 30 s at 95 °C, followed by 24 cycles of amplification (30 sec at 95 °C, 30 sec at 60 °C and 10 min at 72 °C). A final extension step was carried out at 72 °C for 15 min. 1 µL of the restriction enzyme DpnI was added to the PCR mix and incubated at room temperature overnight. *Escherichia coli* MC1061 cells were transformed with this mix and transformants were selected on LB agar plates containing kanamycin (50 µg/ml). The expression plasmids were prepared from an overnight 5 ml LB culture of transformants. The fidelity of all constructs was confirmed by Sanger sequencing. The constructs were introduced into *E. coli* BL21 (DE3) pLysS cells (Novagen) for expression of wild-type Alr_{Mtb} and the three mutants. Transformants were selected on LB agar plates containing kanamycin (50 µg/ml) and chloramphenicol (34 µg/ml).

Expression and purification of Alr_{Mtb}

E. coli BL21 (DE3) pLysS cells containing the wild-type and mutant *alr* constructs were grown in LB medium supplemented with kanamycin (50 µg/ml) and chloramphenicol (34 µg/ml) at 37°C. Overexpression of Alr_{Mtb} protein was induced at OD_{600} of 0.5-0.7 by adding 0.5 mM (final conc.) IPTG and 0.1 mM (final conc.) pyridoxal 5'-phosphate (PLP), and cells were grown overnight. After centrifugation, cells were resuspended and lysed in 20 mM Tris-HCl pH 7.4 buffer containing 500 mM NaCl, 20 mM imidazole and 0.5 mM PLP. The cell lysate was centrifuged and loaded onto a 5 ml HisTrap™ FF crude (GE Healthcare) column equilibrated with a lysis buffer. After the initial washing step, Alr_{Mtb} was eluted with a 20-500 mM imidazole gradient. Alr_{Mtb} fractions were pooled and dialyzed against 20 mM Bicine pH 9.0. The dialyzed sample was then loaded onto a MonoQ™ 5/50 GL (GE Healthcare) and eluted with a 0-500 mM NaCl gradient. Fractions containing $Alr_{Mtb,}$ were further purified using gel filtration chromatography (HiLoad 26/60 Superdex™ 200 prep grade; GE Healthcare) with buffer containing 20 mM Bicine pH 9.0 and 150 mM NaCl. The purity of Alr_{Mtb} samples was analyzed with SDS-PAGE. Protein concentration was determined with Bradford reagent (Thermo Fisher Scientific) as recommended by the manufacturer.

Enzyme assay and determination of D-cycloserine (DCS) IC_{50} for Alr_{Mtb}

The Alr_{Mtb} activity was measured using the *D*-alanine → *L*-alanine coupled reaction as described previously, except for the assay temperature that was changed from 23°C to 30°C.[6] Rates were measured on an Ultrospec 3100 pro with SWIFTII software and analyzed using GraphPad Prism version

6.00 for Windows (GraphPad Software, La Jolla California USA). The DCS inhibition assay was measured at a fixed substrate concentration of 2.5 mM *D*-alanine. The activity of wild-type Alr_{Mtb} and single mutants were measured at various DCS concentrations ranging from 1.4 µM to 1.0 mM. The enzyme concentration used to determine IC_{50} was varied due to the large reduction in the turnover rates of some of Alr mutants. 47 nM enzyme was used for the wild-type and Y364D mutant, and 470 and 235 nM were used for M319T and R373L mutants, respectively. Each reaction mix was pre-incubated with DCS for 30 minutes and the absorbance at 340 nm was measured for 2 minutes to set a baseline. The reaction was started by adding D-alanine to the assay mix and, following equilibration, the rates were measured for 10 minutes. The activity was normalised against a control with no DCS present in the assay mix. Activity assay at each concentration was performed in triplicate.

Structural analysis of Alr_{Mtb} mutations

For the structure-based analysis of the Alr mutations, an Alr_{Mtb}-DCS complex model was created by superposing the crystal structures of *M. tuberculosis* Alr_{Mtb} (PDB: 1XFC) and the *Geobacillus stearothermophilus* Alr adduct with DCS (PDB: 1EPV) (12, 13). PyMOL was used for inspecting the structure and preparing Figure S1 (14). The overall structure of Alr was highly conserved across bacterial species, including the core residues that form the active site (13, 15-19). Indeed 12 out of 13 active site residues were identical between two Alrs used in this model generation. Given a Cα root-mean-square deviation of 0.4 Å for these 13 residues, the two structures were superposed to place the PLP-DCS molecule into the Alr_{Mtb} structure. Although W88 (Alr_{Mtb} numbering) was replaced with leucine in the *G. stearothermophilus* Alr, this single mutation did not influence positioning of the co-factor PLP in both structures.

Ethics approvals

Estonia: The isolate used in this study originates from the strain archive held by the Department of Mycobacteriology, United Laboratories, Tartu University Hospital. Ethical approval for whole-genome sequencing was granted by the Research Ethics Committee of the University of Tartu 219/T-18.

Germany: The molecular epidemiological studies were embedded in mandatory routine surveillance and contact investigation work performed by the public health offices according to the legal mandate of the German Infection Protection Act. They were approved by the Hamburg and Schleswig-Holstein Commissioners for Data Protection.

India: The sequencing received approval from the National Institute for research in Tuberculosis Ethics Committee Chennai, India (NIRT IEC No: 2014002).

Swaziland: Ethical approval was obtained from the Ministry of Health and Social Welfare Scientific Ethical Committee of Swaziland and the Ethics Review Board of MSF.

UK: Approval for whole-genome sequencing and analysis was granted by the UK National Research Ethics Service (12/EE/0439) and the Cambridge University Hospitals NHS Foundation Trust R&D Department (A092685).

Table S1

Overview of the *alr* mutants included in this study with the associated MIC data, where applicable. Details of the study population from Swaziland can be found elsewhere (20, 21). H37Rv was tested as a negative control in all cases. Moreover, four wild-type Portuguese strains were used as controls for the Portuguese *alr* M319T mutants (Figure 1). We also included the baseline isolate PBm0, which developed DCS resistance during treatment (PBm14). Isolates in bold were deemed resistant using the following criteria: MIC>CC=30 µg/ml for the 1% critical proportion method on LJ, MIC>tentative ECOFF=20 µg/ml for the 10% critical proportion method on LJ, and at least a two-fold MIC increase compared with the wild-type controls for MGIT and Sensititre.

		MIC (µg/ml)				Country of				
**Alr mutation**	**Strain**	**1% LJ**	**10% LJ[e]**	**1% MGIT[f]**	**Sensititre[f]**	**isolation**	**Comment**	**Genotype**	**Accession**	**Reference**
**C-8T [a]**	PBm14[b]	**60**[c]				Georgia	XDR	NA	NA	(22)
	PBm20[b]					Georgia	XDR	2.2.1	SRX202647	(22)
	TKK_02_0050		**>60 & >60**			South Africa	XDR	4.3.3	PRJNA190974	(23)
**aTg/aCg M319T[a]**	52149	**40**[d]				India	XDR	2.2.1	ERS1137013	this study
	TKK_04_0105		**>60**			South Africa	XDR	4.3.3	PRJNA235575	(23)
	9440-02					Germany	MDR	2.2.1	ERS1110442	this study
	PT1			**64**	16	Portugal	MDR	4.3.4.2	ERS1534765	this study
	PT5			**64**	32	Portugal	MDR	4.3.4.2	ERS1534769	this study
	PT2			**64**	16	Portugal	XDR	4.3.4.2	ERS1534766	this study
**Tac/Gac Y364D[a]**	52250	**40**[d]				India	XDR	4.1.2.1	ERS1137014	this study
	6844-06					Germany	pre-XDR	2.2.1	ERS457716	this study
	6637-09					Swaziland	MDR	1.2.2	ERS456935	this study
	5558-09					Swaziland	MDR	1.2.2	ERS457677	this study
	6269-09					Swaziland	MDR	4.1.2.1	ERS458618	this study
	117-10					Swaziland	MDR	4.1.2.1	ERS458502	this study
**tAc/tGc Y364C[a]**	159-04					Estonia	MDR	2.2.1	ERS1110440	this study
**cGt/cTt R373L[a]**	50503	**40**[d]				India	pre-XDR	1.1.2	ERS1137015	this study
**Cgt/Ggt R373G[a]**	TKK_02_0007		**25 & 25**			South Africa	XDR	4.3.3	PRJNA190930	(23)
**Wild-type**	H37Rv ATCC 27294	7.5[c] & 10[d]		4	8			NA	NA	
	PBm0[b]	15[c]				Georgia	MDR	2.2.1	SRX202644	(22)
	PT7			16	16	Portugal	INH-R	4.3.4.2	ERS1534771	this study
	PT3			16	8	Portugal	MDR	4.3.4.2	ERS1534767	this study
	PT6			16	16	Portugal	MDR	4.3.4.2	ERS1534770	this study
**ctA/ctG L234L**	PT4			16	8	Portugal	XDR	4.3.4.2	ERS1534768	this study

[a] Correspond to codons 22, 343, 388 and 397 based on the current annotation of H37Rv genome (AL123456.3), which differs from the correct annotation (11).

[b] Isolates from patient B at months 1, 14 and 20 (22).

[c] Tested in Germany.

[d] Tested in India.

[e] Previously tested in South Africa (24).

[f] Tested in Portugal.

Table S2

Overview of mutations in other known or potential DCS resistance genes in 22 isolates from Table S1, for which WGS data were available. The *ald* T-32C, *cycA* R93L and *ddlA* T365A mutations are most likely polymorphisms that do not confer resistance as these occurred in isolates with low as well as high DCS MICs (Table S1).

strain	*ald*	*cycA*	*ddlA*
PBm20	T-32C	R93L (cGg/cTg)	T365A (Aca/Gca)
TKK_02_0050	T-32C	R93L (cGg/cTg)	T365A (Aca/Gca)
52149	T-32C	R93L (cGg/cTg)	T365A (Aca/Gca)
TKK_04_0105	T-32C	R93L (cGg/cTg)	T365A (Aca/Gca)
9440-02	T-32C	R93L (cGg/cTg)	T365A (Aca/Gca)
PT1	T-32C	R93L (cGg/cTg)	T365A (Aca/Gca)
PT5	T-32C	R93L (cGg/cTg)	T365A (Aca/Gca)
PT2	T-32C	R93L (cGg/cTg)	T365A (Aca/Gca)
52250	T-32C	R477G (Cgt/Ggt), R93L (cGg/cTg), L80L (ctg/Ttg)	T365A (Aca/Gca)
6844-06	T-32C	P188A (Cct/Gct), R93L (cGg/cTg)	T365A (Aca/Gca)
6637-09	T-32C	S187P (Tcc/Ccc), R93L (cGg/cTg), C-14T	T365A (Aca/Gca)
5558-09	T-32C	S187P (Tcc/Ccc), R93L (cGg/cTg), C-14T	T365A (Aca/Gca)
6269-09	T-32C, **133 delA**	R477G (Cgt/Ggt), R93L (cGg/cTg), L80L (Ctg/Ttg)	T365A (Aca/Gca)
117-10	T-32C, **133 delA**	R477G (Cgt/Ggt), R93L (cGg/cTg), L80L (Ctg/Ttg)	T365A (Aca/Gca)
159-04	T-32C	R93L (cGg/cTg)	T365A (Aca/Gca)
50503	T-32C, **460 delG**	R93L (cGg/cTg), C-14T	T365A (Aca/Gca)
TKK_02_0007	T-32C	R93L (cGg/cTg)	T365A (Aca/Gca)
PBm0	T-32C	R93L (cGg/cTg)	T365A (Aca/Gca)
PT7	T-32C	R93L (cGg/cTg)	T365A (Aca/Gca)
PT3	T-32C	R93L (cGg/cTg)	T365A (Aca/Gca)
PT6	T-32C	R93L (cGg/cTg)	T365A (Aca/Gca)
PT4	T-32C	R93L (cGg/cTg)	T365A (Aca/Gca)

Table S3

Primers used for site-directed mutagenesis.

Name	Sequence
M319TFwd	5’ GGATCTGC ACGGACCAGTTCATGGTCGACCTGGGCCCC GGG 3’
M319TRev	5’ AACTGGTCCGTGCAGATCCGCCCCACACCGGGGCATCGTCT 3
Y364DFwd	5’ CCATCCACGACGAAGTGGTCACCAGCCCGCGAGGACGTATC 3’
Y364DRev	5’ ACCACTTCGTCGTGGATGGTGCCGACAAGATCGGCCCA GTC 3’
R373LFwd	5’ CGCGAGGACTTATCACCAGGACCTATCGC GAGGCTGAAAAC 3’
R373LRev	5’ CTGGTGATAAGTCCTCGCGG GCTGGTGACCACTTCGTAGTG 3’

Figure S1. Alr$_{Mtb}$-DCS complex model

(A) Overall schematic view of the Alr$_{Mtb}$-DCS complex structure model, including the PLP co-factor. The dimeric Alr$_{Mtb}$ is shown as a ribbon diagram with each monomer of the homodimer colored in light yellow and cyan, respectively. A truncated molecular surface was used to highlight one of the two active sites in the dimer. Some residues were removed for clarity. The left panel shows the entryway of the active site, whereas the view from the opposite side is shown in the right panel. (B) Zoomed-in view of the boxed areas from Panel A. The M319, Y364 and R373 side chains are shown in green. The PLP-DCS molecule is shown in lavender, blue and red. The active site surface model is also shown. Some residues were removed for clarity. (C) Same view as Figure S1B but showing mutated side chains for M319T, Y364D and R373L as purple balls and sticks.

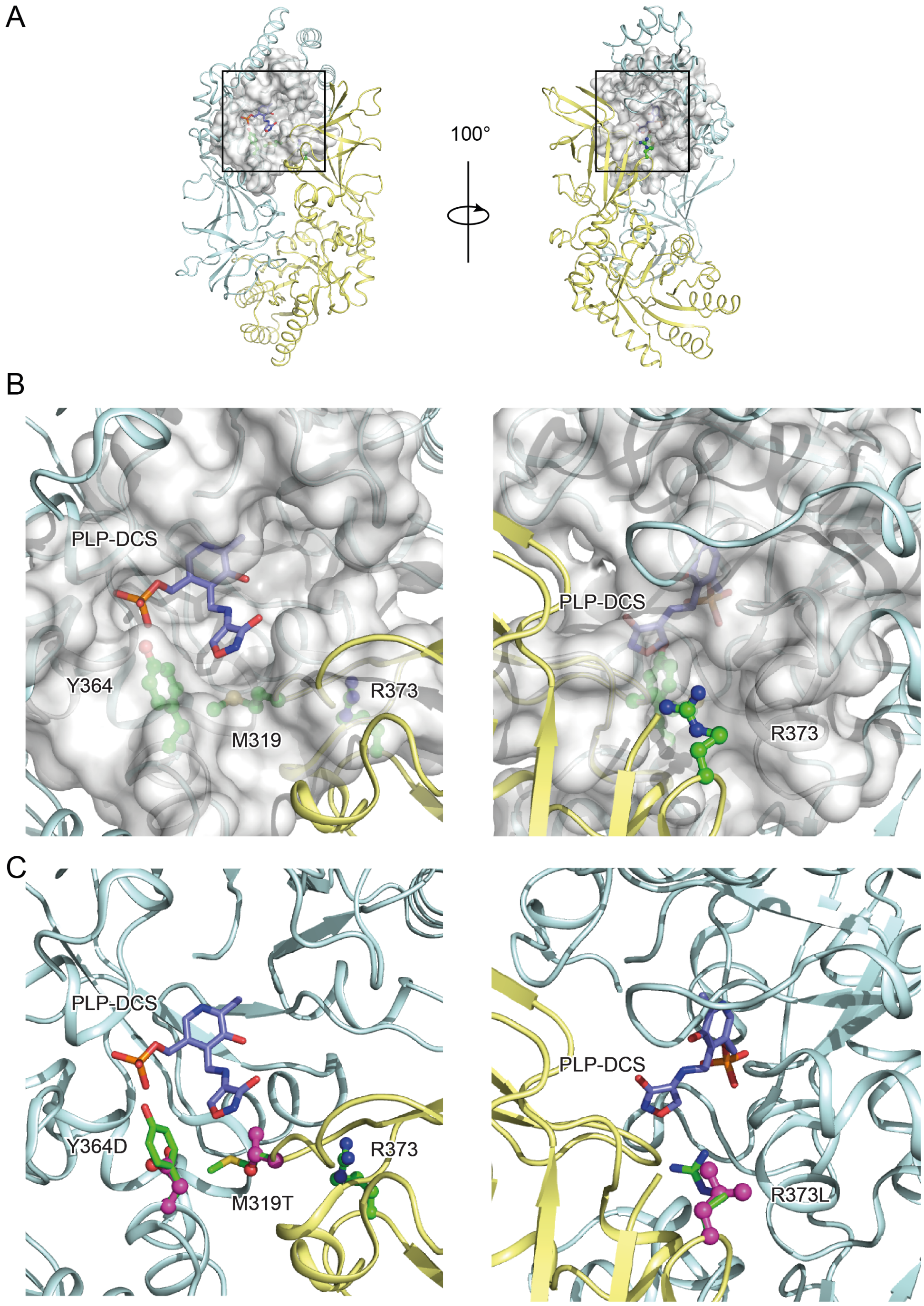

References

1. **Springer B, Lucke K, Calligaris-Maibach R, Ritter C, Böttger EC.** 2009. Quantitative drug susceptibility testing of *Mycobacterium tuberculosis* by use of MGIT 960 and EpiCenter instrumentation. J Clin Microbiol **47:**1773-1780.
2. **Cambau E, Viveiros M, Machado D, Raskine L, Ritter C, Tortoli E, Matthys V, Hoffner S, Richter E, Perez Del Molino ML, Cirillo DM, van Soolingen D, Böttger EC.** 2015. Revisiting susceptibility testing in MDR-TB by a standardized quantitative phenotypic assessment in a European multicentre study. J Antimicrob Chemother **70:**686-696.
3. **Comas I, Coscolla M, Luo T, Borrell S, Holt KE, Kato-Maeda M, Parkhill J, Malla B, Berg S, Thwaites G, Yeboah-Manu D, Bothamley G, Mei J, Wei L, Bentley S, Harris SR, Niemann S, Diel R, Aseffa A, Gao Q, Young D, Gagneux S.** 2013. Out-of-Africa migration and Neolithic coexpansion of *Mycobacterium tuberculosis* with modern humans. Nat Genet **45:**1176-1182.
4. **Coll F, McNerney R, Guerra-Assuncao JA, Glynn JR, Perdigão J, Viveiros M, Portugal I, Pain A, Martin N, Clark TG.** 2014. A robust SNP barcode for typing *Mycobacterium tuberculosis* complex strains. Nat Commun **5:**4812.
5. **Feuerriegel S, Köser CU, Niemann S.** 2014. Phylogenetic polymorphisms in antibiotic resistance genes of the *Mycobacterium tuberculosis* complex. J Antimicrob Chemother **69:**1205-1210.
6. **Li H, Durbin R.** 2009. Fast and accurate short read alignment with Burrows-Wheeler transform. Bioinformatics **25:**1754-1760.
7. **McKenna A, Hanna M, Banks E, Sivachenko A, Cibulskis K, Kernytsky A, Garimella K, Altshuler D, Gabriel S, Daly M, DePristo MA.** 2010. The Genome Analysis Toolkit: a MapReduce framework for analyzing next-generation DNA sequencing data. Genome Res **20:**1297-1303.
8. **Li H, Handsaker B, Wysoker A, Fennell T, Ruan J, Homer N, Marth G, Abecasis G, Durbin R.** 2009. The Sequence Alignment/Map format and SAMtools. Bioinformatics **25:**2078-2079.
9. **Price MN, Dehal PS, Arkin AP.** 2010. FastTree 2-approximately maximum-likelihood trees for large alignments. PLoS One **5:**e9490.
10. **Liu H, Naismith JH.** 2008. An efficient one-step site-directed deletion, insertion, single and multiple-site plasmid mutagenesis protocol. BMC Biotechnol **8:**91.
11. **Strych U, Penland RL, Jimenez M, Krause KL, Benedik MJ.** 2001. Characterization of the alanine racemases from two mycobacteria. FEMS Microbiol Lett **196:**93-98.
12. **Fenn TD, Stamper GF, Morollo AA, Ringe D.** 2003. A side reaction of alanine racemase: transamination of cycloserine. Biochemistry **42:**5775-5783.
13. **LeMagueres P, Im H, Ebalunode J, Strych U, Benedik MJ, Briggs JM, Kohn H, Krause KL.** 2005. The 1.9 Å crystal structure of alanine racemase from *Mycobacterium tuberculosis* contains a conserved entryway into the active site. Biochemistry **44:**1471-1481.
14. **DeLano WL.** PyMOL. http://www.pymol.org/.

15. **LeMagueres P, Im H, Dvorak A, Strych U, Benedik M, Krause KL.** 2003. Crystal structure at 1.45 Å resolution of alanine racemase from a pathogenic bacterium, *Pseudomonas aeruginosa*, contains both internal and external aldimine forms. Biochemistry **42:**14752-14761.
16. **Counago RM, Davlieva M, Strych U, Hill RE, Krause KL.** 2009. Biochemical and structural characterization of alanine racemase from *Bacillus anthracis* (Ames). BMC Struct Biol **9:**53.
17. **Im H, Sharpe ML, Strych U, Davlieva M, Krause KL.** 2011. The crystal structure of alanine racemase from *Streptococcus pneumoniae*, a target for structure-based drug design. BMC Microbiol **11:**116.
18. **Scaletti ER, Luckner SR, Krause KL.** 2012. Structural features and kinetic characterization of alanine racemase from *Staphylococcus aureus* (Mu50). Acta Crystallogr D Biol Crystallogr **68:**82-92.
19. **Davis E, Scaletti-Hutchinson E, Opel-Reading H, Nakatani Y, Krause KL.** 2014. The structure of alanine racemase from *Acinetobacter baumannii*. Acta Crystallogr F Struct Biol Commun **70:**1199-1205.
20. **Sanchez-Padilla E, Dlamini T, Ascorra A, Rüsch-Gerdes S, Tefera ZD, Calain P, de la Tour R, Jochims F, Richter E, Bonnet M.** 2012. High prevalence of multidrug-resistant tuberculosis, Swaziland, 2009-2010. Emerg Infect Dis **18:**29-37.
21. **Sanchez-Padilla E, Merker M, Beckert P, Jochims F, Dlamini T, Kahn P, Bonnet M, Niemann S.** 2015. Detection of drug-resistant tuberculosis by Xpert MTB/RIF in Swaziland. N Engl J Med **372:**1181-1182.
22. **Merker M, Kohl TA, Roetzer A, Truebe L, Richter E, Rüsch-Gerdes S, Fattorini L, Oggioni MR, Cox H, Varaine F, Niemann S.** 2013. Whole genome sequencing reveals complex evolution patterns of multidrug-resistant *Mycobacterium tuberculosis* Beijing strains in patients. PLoS One **8:**e82551.
23. **Cohen KA, Abeel T, Manson McGuire A, Desjardins CA, Munsamy V, Shea TP, Walker BJ, Bantubani N, Almeida DV, Alvarado L, Chapman SB, Mvelase NR, Duffy EY, Fitzgerald MG, Govender P, Gujja S, Hamilton S, Howarth C, Larimer JD, Maharaj K, Pearson MD, Priest ME, Zeng Q, Padayatchi N, Grosset J, Young SK, Wortman J, Mlisana KP, O'Donnell MR, Birren BW, Bishai WR, Pym AS, Earl AM.** 2015. Evolution of extensively drug-resistant tuberculosis over four decades: whole genome sequencing and dating analysis of *Mycobacterium tuberculosis* isolates from KwaZulu-Natal. PLoS Med **12:**e1001880.
24. **Desjardins CA, Cohen KA, Munsamy V, Abeel T, Maharaj K, Walker BJ, Shea TP, Almeida DV, Manson AL, Salazar A, Padayatchi N, O'Donnell MR, Mlisana KP, Wortman J, Birren BW, Grosset J, Earl AM, Pym AS.** 2016. Genomic and functional analyses of *Mycobacterium tuberculosis* strains implicate *ald* in D-cycloserine resistance. Nat Genet **48:**544-551.
